# Therapeutic Potential of Tetrandrine Compared to Temozolomide in Treating Glioblastoma Multiforme Under Normoxic and Hypoxic Conditions

**DOI:** 10.3390/ijms27115090

**Published:** 2026-06-04

**Authors:** Mona A. Khamis, Dalia Abdo, Fatma G. Mohamed, Marwan Emara

**Affiliations:** 1 Center for Aging and Associated Diseases, Zewail City of Science, Technology and Innovation, Giza 12578, Egypt; p-mona.khamis@zewailcity.edu.eg (M.A.K.); p-dalia.mahmoud@zewailcity.edu.eg (D.A.); 2 Chemistry Department (Biochemistry Division), Faculty of Science, Cairo University, Giza 12613, Egypt; fgamal@sci.cu.edu.eg

**Keywords:** glioblastoma multiforme, normoxia, hypoxia, tetrandrine, temozolomide

## Abstract

Glioblastoma multiforme (GBM) is the most aggressive brain tumor in adults. Necrosis, and by inference hypoxia, is a pathognomonic feature of GBM tumors, where hypoxia significantly contributes to chemoresistance, leading to local treatment failure and disease progression. Although temozolomide (TMZ) is the main treatment option, 60–75% of GBM patients do not benefit from it. This study aimed to evaluate the therapeutic potential of Tetrandrine (TET) in combination with or compared to TMZ in treating GBM cells (M010b and U87) under both normoxic and hypoxic conditions. The therapeutic potential was assessed using qRT-PCR, MTT assay, combination index analysis, flow cytometry for apoptosis and cell cycle analysis, scratch assay, gelatin zymography, measurement of mitochondrial membrane potential (ΔΨm), reactive oxygen species (ROS) production, and molecular docking. Under both normoxic and hypoxic conditions, TET showed significant cytotoxicity in both cell lines compared to TMZ. A synergistic effect was observed only under normoxia at 2× IC_50_ concentrations in M010b cells, and at 4× IC_50_ concentrations in U87 cells. TET significantly increased the sub-G1 cell population and apoptosis compared to TMZ in both cell lines under normoxic and hypoxic conditions, while TMZ induced G2/M arrest in U87 cells under both conditions. TET significantly increased ROS production in both cell lines under normoxia. Under both conditions, ΔΨm was significantly reduced by TET in M010b cells and by TMZ in both cell lines. TET and TMZ significantly reduced pro-MMP-2 levels in M010b cells under both conditions and in U87 cells under normoxia. In conclusion, given the limited therapeutic potential of TMZ, our findings suggest that TET could be a viable alternative treatment option for GBM.

## 1. Introduction

Glioblastoma multiforme (GBM), classified by the World Health Organization (WHO) as a grade IV astrocytoma, is the most prevalent intraparenchymal brain tumor with high invasiveness and aggressive clinical characteristics [1,2]. GBM is histologically identified by the presence of pseudopalisades, necrotic foci, and microvascular hyperplasia, primarily mediated by angiogenesis-induced hypoxia, which stimulates the rapid growth of cancer cells [3]. Although the current therapeutic regimen for GBM is dependent on a trimodal approach involving a combination of chemotherapy and radiotherapy after tumor resection, the median survival rate is approximately only 14.6 months [4]. Temozolomide (TMZ), an alkylating agent, is the standard chemotherapy for GBM. TMZ damages tumor cells by forming 6-methylguanine lesions that cause DNA mismatch, resulting in tumor cell senescence, autophagy, and apoptosis [5]. Several mechanisms contribute to TMZ resistance, including tumor-intrinsic factors such as DNA repair via O^6^-methylguanine-DNA methyltransferase (MGMT) and tumor microenvironment-mediated processes, encompassing immune evasion mechanisms and hypoxia-induced signaling pathways [6]. A high-dose regimen is required to achieve effective concentrations in the target tissues, but its administration can provoke serious systemic side effects, such as hematological and gastrointestinal toxicities, without improving the risk/benefit ratio [6,7]. Moreover, for newly diagnosed GBM patients, there was only a 2.5-month increase in the median survival following treatment with TMZ and radiotherapy compared with radiotherapy alone. In addition, 60–75% of patients with GBM get no benefit from TMZ treatment, and more than 50% of GBM patients with recurrent anaplastic gliomas fail TMZ treatment [8] that often leads to non-specific cytotoxicity [9]. Altogether, these suggest a modest therapeutic efficacy of TMZ, which warrants a search for new alternatives in GBM chemotherapy.

Hypoxia results from the rapid proliferation of tumor cells and unorganized tumor neovascularization, leading to reduced oxygen uptake [10]. It was reported that the physiological oxygen concentration in healthy brain tissues is between 12.5% and 2.5%; however, it ranges from 0.1% to 2.5% in glioma masses [11,12]. Accordingly, tumor cells adapt to the hypoxic microenvironment through a transcriptional program regulated by hypoxia-inducible factor (HIF-1) to prevent apoptosis [13]. Under hypoxic conditions, the HIFα subunit is translocated to the nucleus, where it binds to hypoxia-responsive elements (HREs) in the promoter regions of over 100 downstream target genes that regulate glycolysis, angiogenesis, apoptosis, cell proliferation, migration, cell survival, and drug resistance [13,14]. It has been shown that overexpression of hypoxia markers, including lactate dehydrogenase A (LDHA), carbonic anhydrase IX (CAIX), vascular endothelial growth factor (VEGF), and glucose transporter 1(GLUT-1), in glioma tissues correlates with dismal prognosis [14,15]. The effect of anticancer drugs on DNA damage is more permanent under normoxia because hypoxic cells can undergo a high degree of restoration [16]. In addition, the mechanism of action of many chemotherapeutic drugs is oxygen-dependent; therefore, chemoresistance is exacerbated under hypoxic conditions [17]. Consequently, it is crucial to determine the cytotoxicity of anti-cancer drugs under hypoxic conditions.

Tetrandrine (TET), a lipid-soluble bisenzylisoquinoline alkaloid isolated from Chinese herbal medicine (*Stephania tetrandra S. Moore*), has been reported to have several pharmacological properties, including anti-inflammatory, anti-oxidative, and anti-hypertensive activities [18]. Increasing evidence suggests that TET could be a potential anticancer treatment as it inhibits proliferation and induces apoptosis by affecting numerous signaling pathways in multiple malignancies [19,20,21,22,23]. Additionally, TET has been shown to reverse P-gp expression, improve drug transport across the blood–brain barrier, and inhibit drug resistance [24,25,26]. TET has also been reported to boost the production of free radicals, increase autophagy, halt the progression of the cell cycle, and suppress angiogenesis, migration, and invasion [27,28,29]. Furthermore, TET sensitizes cancer cells to various chemotherapeutics by enhancing their synergistic effects [24,30,31,32]. Moreover, it was reported that TET at therapeutic doses did not exhibit cytotoxicity in normal cells [26,33,34,35,36,37]. Overall, this suggests that TET is not only an adjuvant to chemotherapy but also a promising alternative anticancer candidate.

This study aimed to evaluate the therapeutic potential of TET in combination with or compared to TMZ in treating GBM cells (M010b and U87) under both normoxic and hypoxic conditions.

## 2. Results

### 2.1. The Effect of Treatment with TET, TMZ, and Their Combination on Cell Viability and Proliferation of GBM Cells Under Normoxic and Hypoxic Conditions

#### 2.1.1. Induction of Hypoxia with CoCl_2_

**Figure 1:**
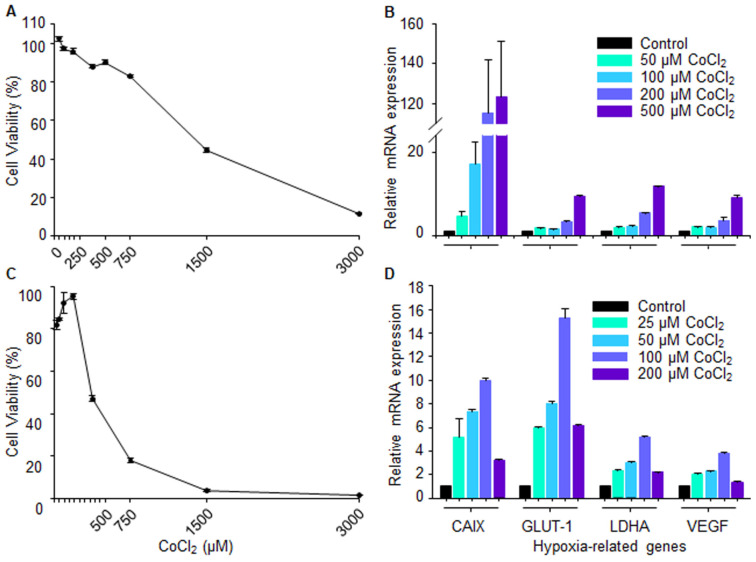
Cell viability and proliferation assay (MTT) of M010b cells (**A**) and U87 cells (**C**) treated with different concentrations of CoCl_2_ for 24 h. The mRNA expression of hypoxia markers (VEGF, CAIX, LDHA, and GLUT1) was measured in M010b cells (**B**) and U87 cells (**D**) by qRT-PCR after exposure to various concentrations of CoCl_2_. Data are presented as fold increase relative to control.

The hypoxia-mimetic agent CoCl_2_ was used to induce hypoxia. The stability of HIF-1α/2α lasts for several hours after CoCl_2_ treatment, with a peak at 12–48 h [38]. To determine the hypoxia-inducing concentrations of CoCl_2_ with minimal cytotoxicity, M010b and U87 cells were exposed to different concentrations of CoCl_2_ for 24 h. The MTT results showed that CoCl_2_ reduced cell viability in a concentration-dependent manner in both M010b (Figure 1A) and U87 cells (Figure 1C). The expression of hypoxia markers (VEGF, CAIX, LDHA, and GLUT1) was measured using qRT-PCR after treating M010b and U87 cells with CoCl_2_ at concentrations of 50–500 μM and 25–100 μM, respectively. In both cell lines, CoCl_2_ treatment increased hypoxia marker levels in a dose-dependent manner (Figure 1B,D). Consequently, CoCl_2_ concentrations of 100 μM and 500 μM were selected for U87 and M010b cells, respectively, to induce the maximum hypoxic responses with minimal cytotoxicity.

#### 2.1.2. Treatment with TET and TMZ

**Figure 2:**
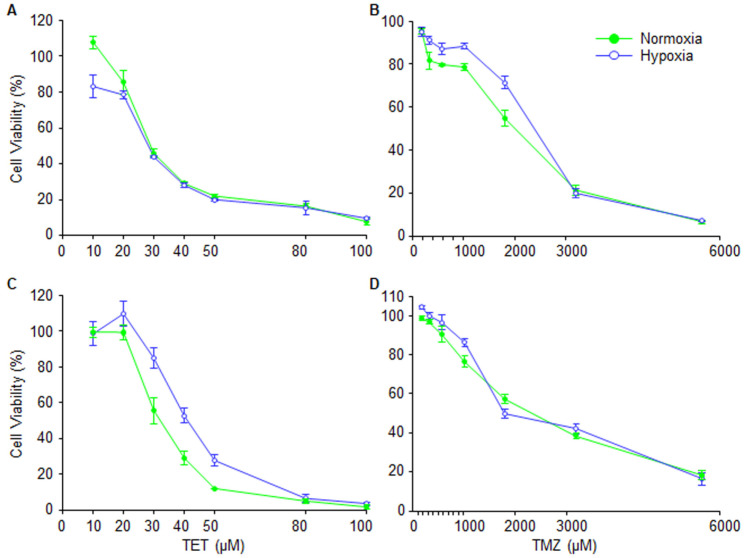
Cell viability and proliferation assay (MTT) of GBM cells treated with various concentrations of TET and TMZ under normoxic and hypoxic conditions for 24 h. (**A**) M010b cells treated with TET; (**B**) M010b cells treated with TMZ; (**C**) U87 cells treated with TET; (**D**) U87 cells treated with TMZ.

**Figure 3:**
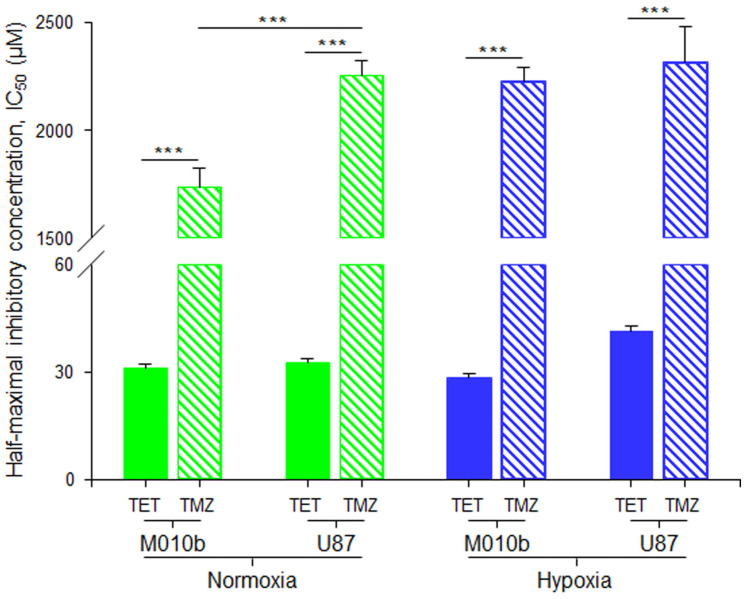
Comparison of the IC_50_ values for TET and TMZ in M010b and U87 cells under normoxic and hypoxic conditions after 24 h. Two-way ANOVA, followed by all pairwise multiple comparisons, was conducted to compare the IC_50_ values of TET and TMZ in both M010b and U87 cells under normoxic (green bars) and hypoxic (blue bars) conditions. *** *p* ≤ 0.001.

Treating M010b (Figure 2A,B) and U87 cells (Figure 2C,D) with TET (10–100 μM) and TMZ (100–5000 μM) for 24 h resulted in a concentration-dependent decrease in cell growth and proliferation in both cell lines under normoxic and hypoxic conditions. The IC_50_ values (Table 1) of TET in both cell lines under both conditions were significantly lower than those of TMZ (*p* ≤ 0.001, two-way ANOVA), indicating that GBM cells are more sensitive to TET than to TMZ (Figure 3). Under normoxia (Figure 3), M010b cells showed significantly higher sensitivity (lower IC_50_) to TMZ compared to U87 cells (*p* ≤ 0.001, two-way ANOVA). However, there was no significant difference between M010b and U87 cells in their response to TET under the same conditions (two-way ANOVA). Under hypoxia (Figure 3), no significant differences were observed between the two cell lines in their responses to either TET or TMZ (two-way ANOVA). Unlike the significant difference in response to TMZ (*p* ≤ 0.01, three-way ANOVA), there were no significant differences between normoxic and hypoxic conditions in the responses of both cell lines to TET.

#### 2.1.3. Treatment with TET and TMZ Combination

**Figure 4:**
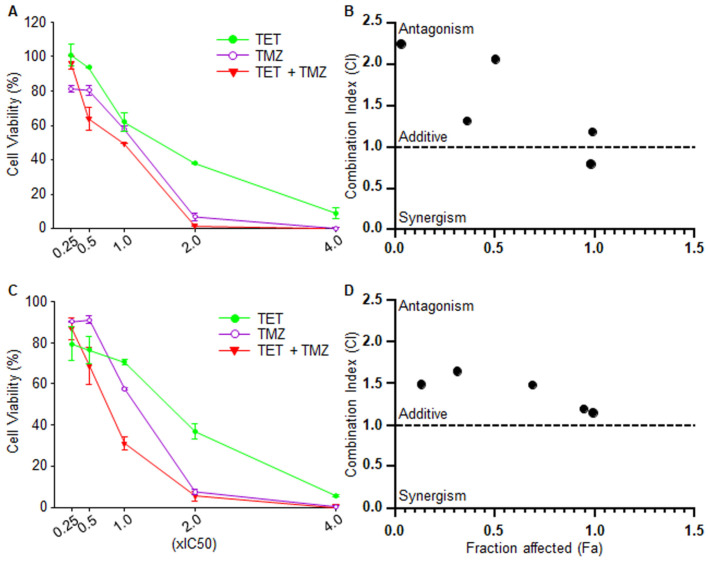
The potency of five IC_50_ ratios (4×, 2×, 1×, 0.5×, and 0.25× IC_50_) for TET and TMZ and their combination for 24 h in M010b cells under normoxic (**A**) and hypoxic (**C**) conditions. The fraction affected (Fa) was calculated from the viability values (%) and used to calculate the combination index (CI) values under normoxia (**B**) and hypoxia (**D**).

**Figure 5:**
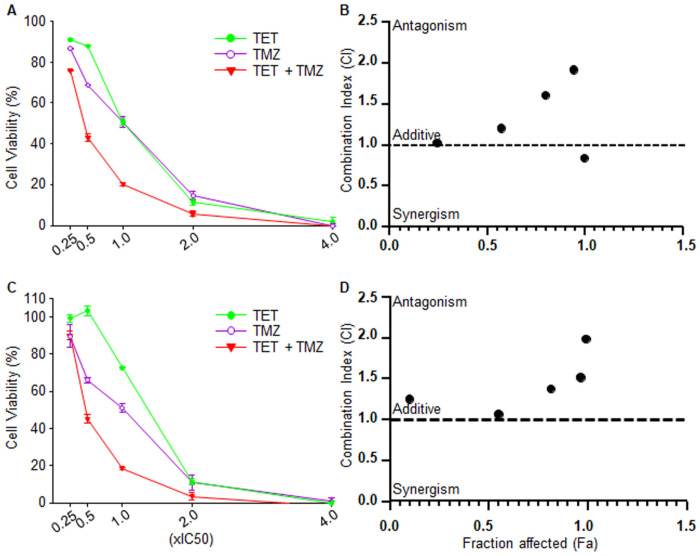
The potency of five IC_50_ ratios (4×, 2×, 1×, 0.5×, and 0.25× IC_50_) for TET and TMZ and their combination for 24 h in U87 cells under normoxic (**A**) and hypoxic (**C**) conditions. The fraction affected (Fa) was calculated from the viability values (%) and used to calculate the combination index (CI) values under normoxia (**B**) and hypoxia (**D**).

Using the combination index analysis described by Chou and Talalay’s method [39], we further examined whether TET could sensitize M010b and U87 cells to TMZ. The potency of TET, TMZ, and their combination at five constant ratios of IC_50_ (4×, 2×, 1×, 0.5×, and 0.25× IC_50_) was tested for 24 h under normoxic and hypoxic conditions in M010b cells (Figure 4A,C) and U87 cells (Figure 5A,C). Under normoxia, in M010b cells, a combination of 2× IC_50_ of TET and TMZ showed synergistic effects (CI = 0.8) (Figure 4B). In U87 cells, a combination of 4× IC_50_ of TET and TMZ, and another combination of 0.25× IC_50_ TET and TMZ resulted in synergistic (CI = 0.84) and additive effects, respectively (Figure 5B). All other tested combinations in both cell lines were antagonistic. Under hypoxia, all tested combinations in both cell lines were antagonistic (Figure 4D and Figure 5D). Consequently, in all subsequent assays, only single treatments at 1× IC_50_ were used, unless otherwise stated.

### 2.2. Cell Cycle Analysis Following Treatment with TET and TMZ

**Figure 6:**
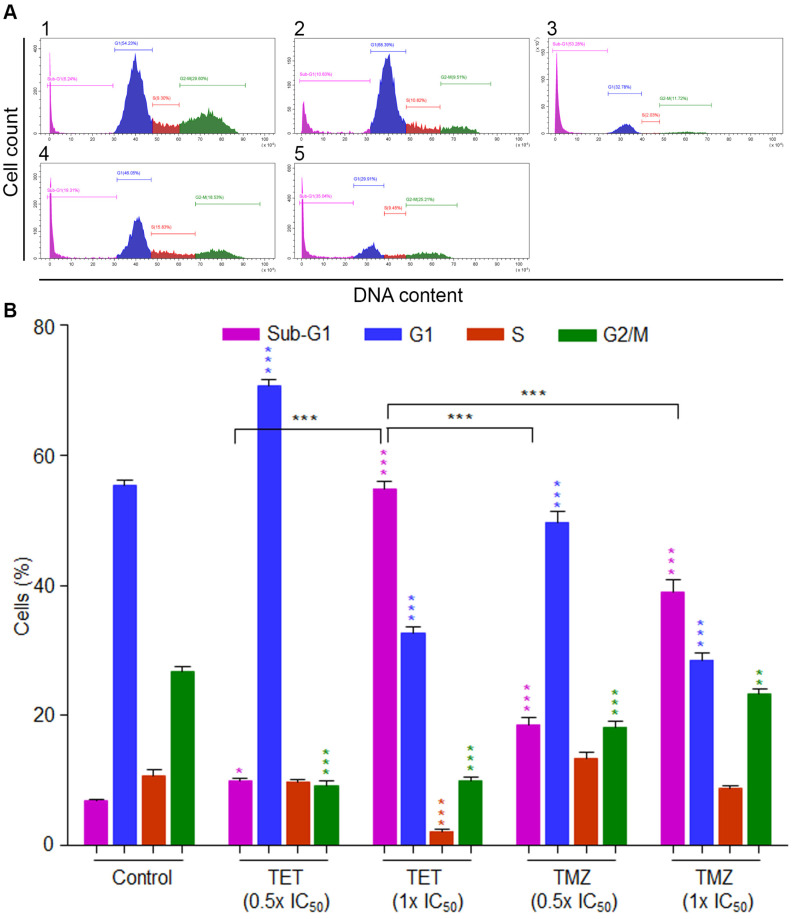
Cell cycle analysis of M010b cells under normoxic conditions following 24 h treatment with 0.5× IC_50_ TET (**A2**,**B**), 1× IC_50_ TET (**A3**,**B**), 0.5× IC_50_ TMZ (**A4**,**B**), and 1× IC_50_ TMZ (**A5**,**B**), alongside control cells (**A1**,**B**). (**A**) Representative histograms showing the fluorescence intensity of propidium iodide (PI) that stains the DNA content at different cell cycle phases. (**B**) Bar chart showing the percentage of cells in the sub-G1 region, and different cell cycle phases (G0/G1, S, and G2/M) following different treatments. One-way ANOVA followed by multiple comparisons versus control was conducted to compare individual cell cycle phases across treatments with their respective controls. Further analysis (one-way ANOVA, followed by all pairwise multiple comparisons) was conducted to compare the percentage of cells in the sub-G1 in control and treated cells (horizontal lines with asterisks). * *p* ≤ 0.05, ** *p* ≤ 0.01, and *** *p* ≤ 0.001.

**Figure 7:**
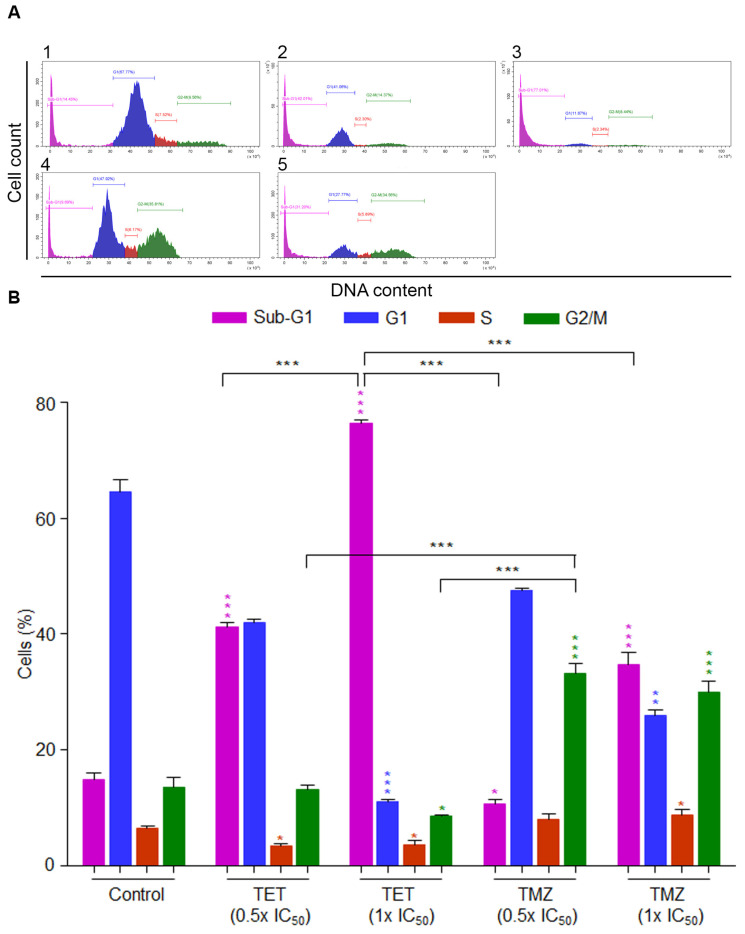
Cell cycle analysis of M010b cells under hypoxic conditions following 24 h treatment with 0.5× IC_50_ TET (**A2**,**B**), 1× IC_50_ TET (**A3**,**B**), 0.5× IC_50_ TMZ (**A4**,**B**), and 1× IC_50_ TMZ (**A5**,**B**), alongside control cells (**A1**,**B**). (**A**) Representative histograms showing the fluorescence intensity of propidium iodide (PI) that stains the DNA content at different cell cycle phases. (**B**) Bar chart showing the percentage of cells in the sub-G1 region, and different cell cycle phases (G0/G1, S, and G2/M) following different treatments. One-way ANOVA followed by multiple comparisons versus control was conducted to compare individual cell cycle phases across treatments and their respective controls. Further analysis (one-way ANOVA followed by all pairwise multiple comparisons) was conducted to compare the percentages of cells in the sub-G1 region or G2/M phase in control and treated cells (horizontal lines with asterisks). * *p* ≤ 0.05, ** *p* ≤ 0.01, and *** *p* ≤ 0.001.

The quantitative analysis of the cell cycle and cell cycle arrests at different phases is essential for understanding how cells respond to treatments under various conditions [40]. As shown in Figure 6, under normoxia, M010b cells exhibited a significant increase in the sub-G1 cell population after treatment with 0.5× IC_50_ TET (9.88%), 1× IC_50_ TET (54.83%), 0.5× IC_50_ TMZ (18.57%), and 1× IC_50_ TMZ (38.92%). The significant accumulation of cells in the sub-G1 region caused by TET or TMZ may indicate apoptotic cell death [41,42]. In the G1 phase, a significant increase was observed with 0.5× IC_50_ TET (70.62%), whereas 1× IC_50_ TET (32.69%), 0.5× IC_50_ TMZ (49.63%), and 1× IC_50_ TMZ (28.50%) resulted in significant decreases. A significant decrease (2.20%) in S phase was observed only in 1× IC_50_ TET treatment. In G2/M phase, significant reductions were observed following treatment with 0.5× IC_50_ TET (9.19%), 1× IC_50_ TET (9.99%), 0.5× IC_50_ TMZ (18.21%), and 1× IC_50_ TMZ (23.34%). Under hypoxia (Figure 7), M010b cells showed a significant increase in the sub-G1 cell population following treatment with 0.5× IC_50_ TET (41.14%), 1× IC_50_ TET (76.30%), and 1× IC_50_ TMZ (34.83%). In contrast, there was a significant decrease in the G1 phase after treatment with 1× IC_50_ TET (11.11%) and 1× IC_50_ TMZ (26.01%). The S phase was significantly decreased following treatment with 0.5× IC_50_ TET (3.51%) and 1× IC_50_ TET (3.69%), while 1× IC_50_ TMZ caused a significant increase (8.77%). Significant increases in the G2/M phase were observed after treatment with 0.5× IC_50_ TMZ (33.21%) and 1× IC_50_ TMZ (30.02%). Conversely, 1× IC_50_ TET resulted in a significant decrease (8.70%). It is noteworthy that under both normoxic and hypoxic conditions (Figure 6 and Figure 7), 1× IC_50_ TET significantly induced the highest increase in sub-G1 cell population compared to control cells, 0.5× IC_50_ TET, 0.5× IC_50_ TMZ, and 1× IC_50_ TMZ (One-Way ANOVA, all pairwise multiple comparisons). Additionally, under hypoxic conditions (Figure 7), 0.5× IC_50_ TMZ significantly induced the highest cell cycle arrest in the G2/M phase compared to control cells, 0.5× IC_50_ TET, and 1× IC_50_ TET (One-Way ANOVA, all pairwise multiple comparisons).

**Figure 8:**
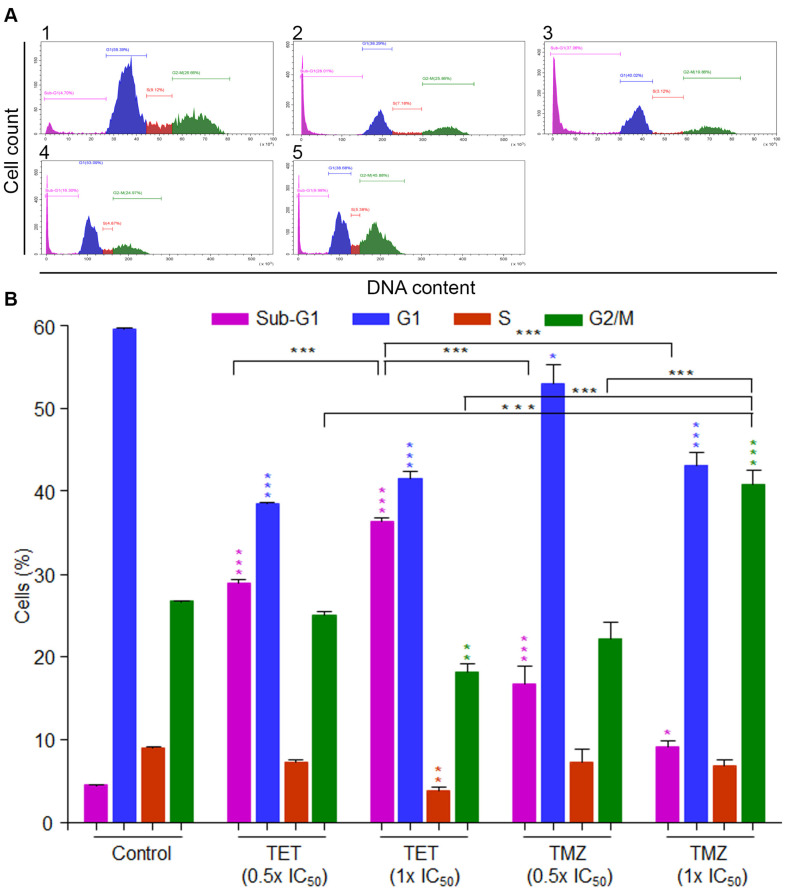
Cell cycle analysis of U87 cells under normoxic conditions following 24 h treatment with 0.5× IC_50_ TET (**A2**,**B**), 1× IC_50_ TET (**A3**,**B**), 0.5× IC_50_ TMZ (**A4**,**B**), and 1× IC_50_ TMZ (**A5**,**B**), alongside control cells (**A1**,**B**). (**A**) Representative histograms showing the fluorescence intensity of propidium iodide (PI) that stains the DNA content at different cell cycle phases. (**B**) Bar chart showing the percentage of cells in the sub-G1 region, and different cell cycle phases (G0/G1, S, and G2/M) following different treatments. One-way ANOVA followed by multiple comparisons versus control was conducted to compare individual cell cycle phases across treatments with their respective controls. Further analysis (one-way ANOVA followed by all pairwise multiple comparisons) was conducted to compare the percentages of cells in the sub-G1 region or G2/M phase in control and treated cells (horizontal lines with asterisks). * *p* ≤ 0.05, ** *p* ≤ 0.01, and *** *p* ≤ 0.001.

**Figure 9:**
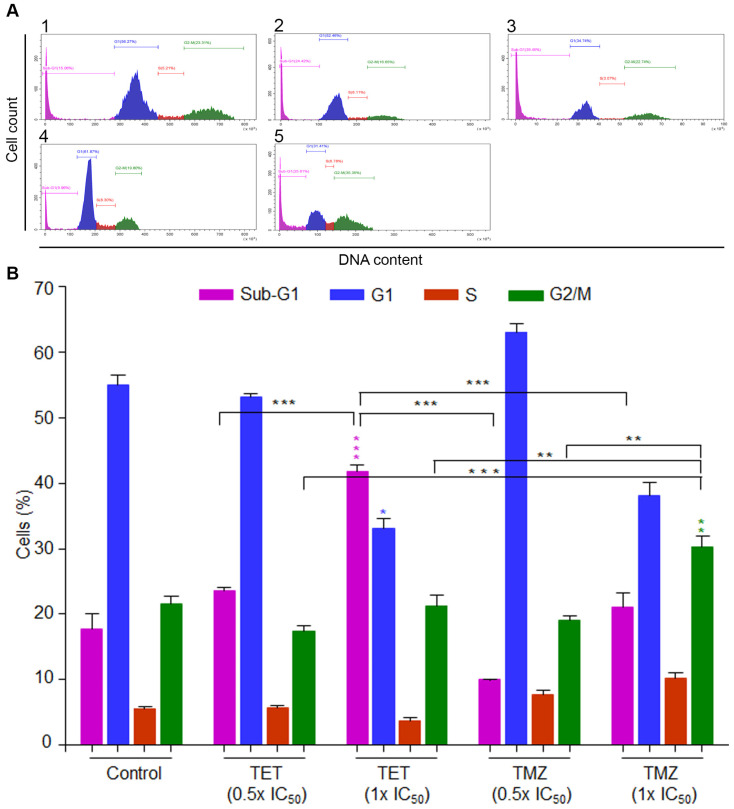
Cell cycle analysis of U87 cells under hypoxic conditions following 24 h treatment with 0.5× IC_50_ TET (**A2**,**B**), 1× IC_50_ TET (**A3**,**B**), 0.5× IC_50_ TMZ (**A4**,**B**), and 1× IC_50_ TMZ (**A5**,**B**), alongside control cells (**A1**,**B**). (**A**) Representative histograms showing the fluorescence intensity of propidium iodide (PI) that stains the DNA content at different cell cycle phases. (**B**) Bar chart showing the percentage of cells in the sub-G1 region, and different cell cycle phases (G0/G1, S, and G2/M) following different treatments. One-way ANOVA followed by multiple comparisons versus control was conducted to compare individual cell cycle phases across treatments and their respective controls. Further analysis (one-way ANOVA followed by all pairwise multiple comparisons) was conducted to compare the percentages of cells in the sub-G1 region or G2/M phase in control and treated cells (horizontal lines with asterisks). * *p* ≤ 0.05, ** *p* ≤ 0.01, and *** *p* ≤ 0.001.

In U87 cells, under normoxic conditions (Figure 8), a significant increase in the sub-G1 cell population was observed after treatment with 0.5× IC_50_ TET (28.91%), 1× IC_50_ TET (36.31%), 0.5× IC_50_ TMZ (16.74%), and 1× IC_50_ TMZ (9.13%). Conversely, a significant decrease in G1 phase was observed following treatment with 0.5× IC_50_ TET (38.50%), 1× IC_50_ TET (41.50%), 0.5× IC_50_ TMZ (52.96%), and 1× IC_50_ TMZ (43.11%). A significant decrease in the S phase was induced upon treatment with 1× IC_50_ TET (3.87%). Additionally, a significant reduction in G2/M phase was induced by 1× IC_50_ TET (18.19%), whereas 1× IC_50_ TMZ caused a significant increase (40.79%). Under hypoxia (Figure 9), treatment with 1× IC_50_ TET resulted in a significant increase in the sub-G1 cell population (41.76%) and a significant decrease in the G1 phase (33.05%). A significant induction in G2/M (30.29%) was seen following treatment with 1× IC_50_ TMZ. Similar to the response observed in M010b cells, in U87 cells (Figure 8 and Figure 9), under both normoxic and hypoxic conditions, 1× IC_50_ TET significantly induced the highest increase in the sub-G1 cell population compared to control cells, 0.5× IC_50_ TET, 0.5× IC_50_ TMZ, and 1× IC_50_ TMZ (One Way ANOVA, all pairwise multiple comparisons). While 1× IC_50_ TMZ (Figure 8 and Figure 9) significantly induced the highest cell cycle arrest in the G2/M phase compared to control cells, 0.5× IC_50_ TET, 1× IC_50_ TET, and 0.5× IC_50_ TMZ under both conditions (One Way ANOVA, all pairwise multiple comparisons).

### 2.3. Apoptosis Is Induced in M010b Cells and U87 Cells by TET and TMZ

**Figure 10:**
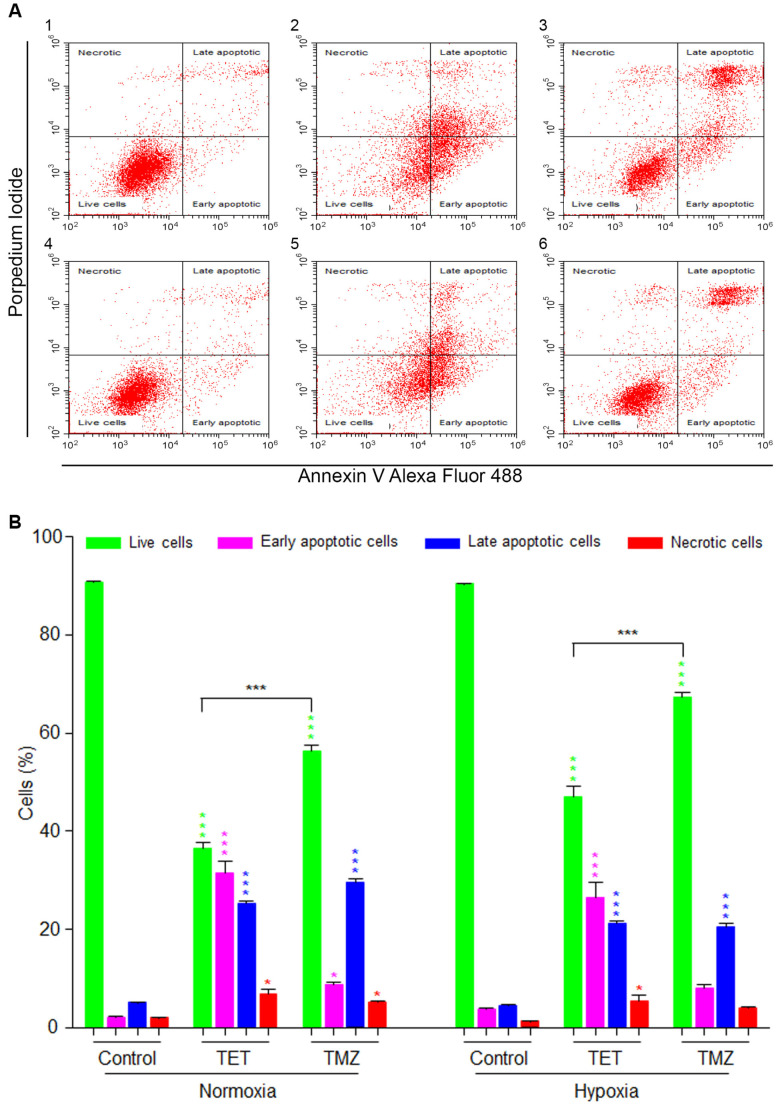
Apoptosis analysis in M010b cells following 24 h treatment with 1× IC_50_ TET and 1× IC_50_ TMZ, under normoxic and hypoxic conditions, alongside control cells. (**A**) Representative flow cytometry dot plots showing live cells, early apoptosis, late apoptosis, and necrosis under normoxic conditions in control cells (**A1**,**B**), TET-treated cells (**A2**,**B**), and TMZ-treated cells (**A3**,**B**), and under hypoxia in control cells (**A4**,**B**), TET-treated cells (**A5,B**), and TMZ-treated cells (**A6**,**B**). (**B**) Percentage of live cells, early apoptosis, late apoptosis, and necrosis in control and treated cells under normoxic and hypoxic conditions. One-way ANOVA, followed by multiple comparisons versus control, was performed to compare live cells, early apoptosis, late apoptosis, and necrosis in different treatments and their respective controls under normoxic and hypoxic conditions (percent bar chart). Further analysis (one-way ANOVA followed by all pairwise multiple comparisons) was conducted to compare the percentage of live cells in control cells, TET-treated cells, and TMZ-treated cells (horizontal lines with asterisks) under normoxia and hypoxia. * *p* ≤ 0.05, and *** *p* ≤ 0.001.

The accumulation of cells in the sub-G1 region may indicate apoptosis, but it is not exclusive [41,42]; therefore, annexin V/PI staining was used to determine whether the induced cell death was mediated by apoptosis or necrosis. Under normoxia (Figure 10), significant decreases in live cells were observed in M010b cells following treatment with TET (36.50%) and TMZ (56.31%). Concomitantly, there were significant increases in early apoptosis, late apoptosis, and necrosis upon treatment with TET and TMZ. Similarly, under hypoxia (Figure 10), TET and TMZ caused significant decreases in live cells by 46.91% and 67.39%, respectively. Significant increases in early apoptosis, late apoptosis, and necrosis were observed following treatment with TET, while TMZ caused a significant increase in late apoptotic cells.

**Figure 11:**
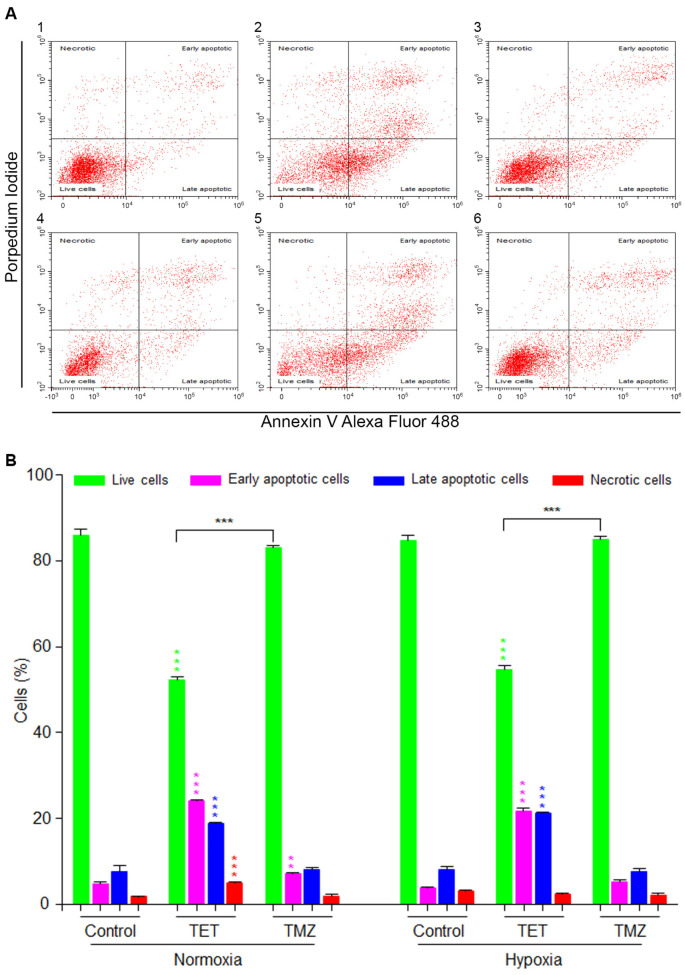
Apoptosis analysis in U87 cells following 24 h treatment with 1× IC_50_ TET and 1× IC_50_ TMZ, under normoxic and hypoxic conditions, alongside control cells. (**A**) Representative flow cytometry dot plots showing live cells, early apoptosis, late apoptosis, and necrosis under normoxic conditions in control cells (**A1**,**B**), TET-treated cells (**A2**,**B**), and TMZ-treated cells (**A3**,**B**), and under hypoxia in control cells (**A4**,**B**), TET-treated cells (**A5**,**B**), and TMZ-treated cells (**A6**,**B**). (**B**) Percentage of live cells, early apoptosis, late apoptosis, and necrosis in control and treated cells under normoxic and hypoxic conditions. One-way ANOVA, followed by multiple comparisons versus control, was performed to compare live cells, early apoptosis, late apoptosis, and necrosis in different treatments and their respective controls under normoxic and hypoxic conditions (percent bar chart). Further analysis (one-way ANOVA followed by all pairwise multiple comparisons) was conducted to compare the percentage of live cells in control cells, TET-treated cells, and TMZ-treated cells (horizontal lines with asterisks) under normoxia and hypoxia. ** *p* ≤ 0.01 and *** *p* ≤ 0.001.

In U87 cells, under normoxia (Figure 11), TET treatment caused a significant decrease (52.25%) in live cells, along with significant increases in early apoptosis (23.97%), late apoptosis (18.77%), and necrosis (5.01%). In contrast, TMZ caused a significant increase (7.13%) in the early apoptotic cells. Under hypoxic conditions (Figure 11), unlike TMZ, TET induced a significant decrease (54.76%) in live cells, along with significant increases in early apoptosis (21.71%) and late apoptosis (21.12%). In both cell lines under both normoxic and hypoxic conditions, TET significantly induced a greater reduction in live cells compared with TMZ (One-Way ANOVA, all pairwise multiple comparisons) (Figure 10 and Figure 11).

### 2.4. Mitochondrial Membrane Potential Response to TET and TMZ Treatments

**Figure 12:**
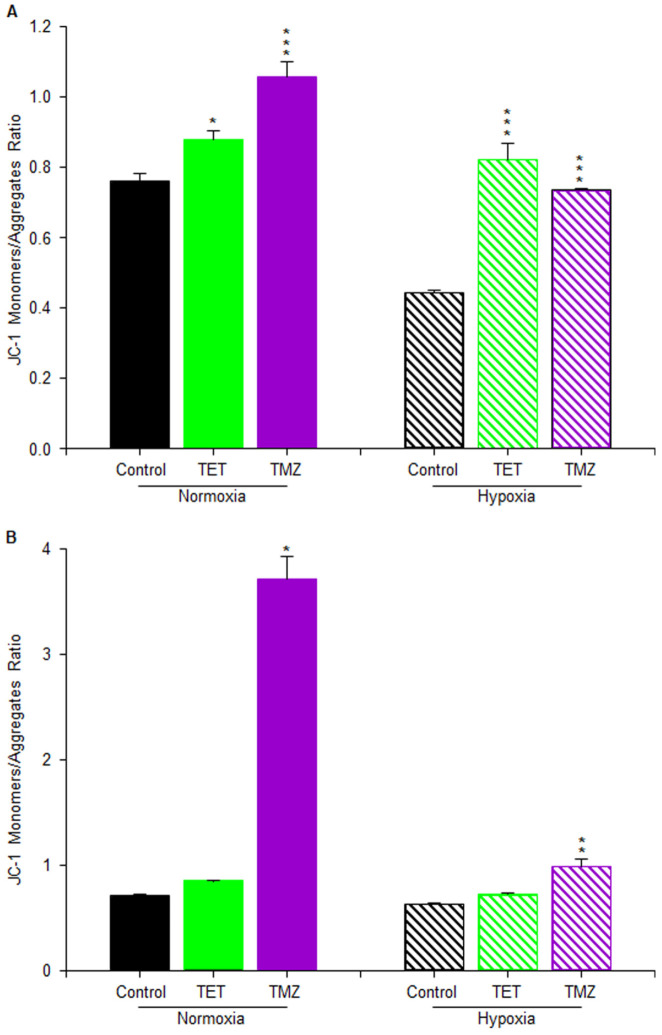
Mitochondrial membrane potential (ΔΨm) evaluated by JC-1 dye and expressed as JC-1 monomer (green fluorescence) to aggregate (red fluorescence) ratio, where a high JC-1 green to red fluorescence ratio is an indication of a decrease in ΔΨm. (**A**) M010b cells were treated with 1× IC_50_ TET and 1× IC_50_ TMZ for 24 h under normoxic and hypoxic conditions, alongside control cells. Following treatments, cells were stained with the JC-1 solution. (**B**) U87 cells were treated with 1× IC_50_ TET and 1× IC_50_ TMZ for 24 h under normoxic and hypoxic conditions, alongside control cells. Then, cells were stained with the JC-1 solution. One-way ANOVA, followed by multiple comparisons versus control, was performed to compare ΔΨm in treated and control cells under normoxic and hypoxic conditions. * *p* ≤ 0.05, ** *p* ≤ 0.01, and *** *p* ≤ 0.001.

JC-1 dye is commonly used to study ΔΨm. It accumulates inside mitochondria, where its shift from red-fluorescent aggregates to green-fluorescent monomers indicates depolarization of the ΔΨm. A decrease in ΔΨm signals oxidative stress and serves as a marker for early apoptotic events. As shown in Figure 12A,B, TET reduced the ΔΨm to 1.16-fold and 1.85-fold of the control in M010b cells under normoxic and hypoxic conditions, respectively. In contrast, the decrease in mitochondrial membrane potential in U87 cells was not significant under either condition. Treatment of M010b cells (Figure 12A) with TMZ significantly reduced ΔΨm to 1.39-fold and 1.56-fold of the control under normoxic and hypoxic conditions, respectively. Similarly, in U87 cells (Figure 12B), it caused significant decreases of 5.23-fold and 1.56-fold in ΔΨm under normoxic and hypoxic conditions, respectively.

### 2.5. Levels of ROS upon Treatment with TET and TMZ

**Figure 13:**
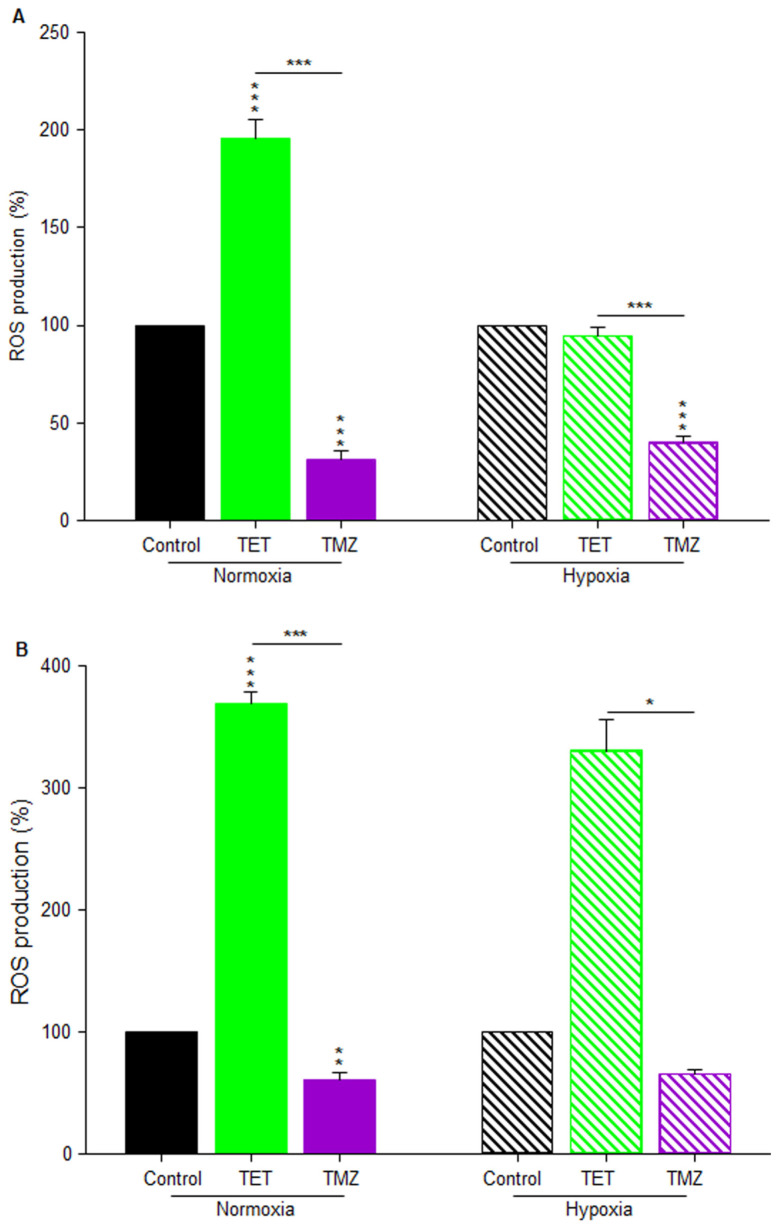
Reactive oxygen species (ROS) production, where CM-H2DCFDA staining was used to measure the intracellular ROS levels. (**A**) M010b cells were treated with 1× IC_50_ TET and 1× IC_50_ TMZ for 24 h under normoxic and hypoxic conditions, alongside control cells. (**B**) U87 cells were treated with 1× IC_50_ TET and 1× IC_50_ TMZ for 24 h under normoxic and hypoxic conditions, alongside control cells. One-way ANOVA, followed by multiple comparisons versus control, was performed to compare the percentages of ROS in treated and control cells under normoxic and hypoxic conditions. Further analysis (one-way ANOVA followed by all pairwise multiple comparisons) was conducted to compare ROS production percentages in control, TET-treated, and TMZ-treated cells (horizontal lines with asterisks) in M010b and U87 cells under normoxia and hypoxia. * *p* ≤ 0.05, ** *p* ≤ 0.01, and *** *p* ≤ 0.001.

The dual role of ROS in cancer is concentration-dependent. At low and moderate levels, ROS promote cancer progression, whereas higher levels, exceeding the cytotoxic threshold, lead to cancer cell death [43]. To assess ROS levels following TET and TMZ treatment, the ROS indicator CM-H2DCFDA was used. As shown in Figure 13A,B, treatment with TET significantly increased ROS production in M010b and U87 cells by 195.68% and 368.49%, respectively, under normoxia. However, no significant effect on ROS levels was observed in either cell line under hypoxia. Interestingly, TMZ significantly decreased ROS levels in M010b under both conditions and in U87 under normoxia. Under both conditions, TET significantly induced greater ROS production in both cell lines compared with TMZ (One-Way ANOVA, all pairwise multiple comparisons) (Figure 13A,B).

### 2.6. TET and TMZ Reduced Cell Migration Following Treatment

**Figure 14:**
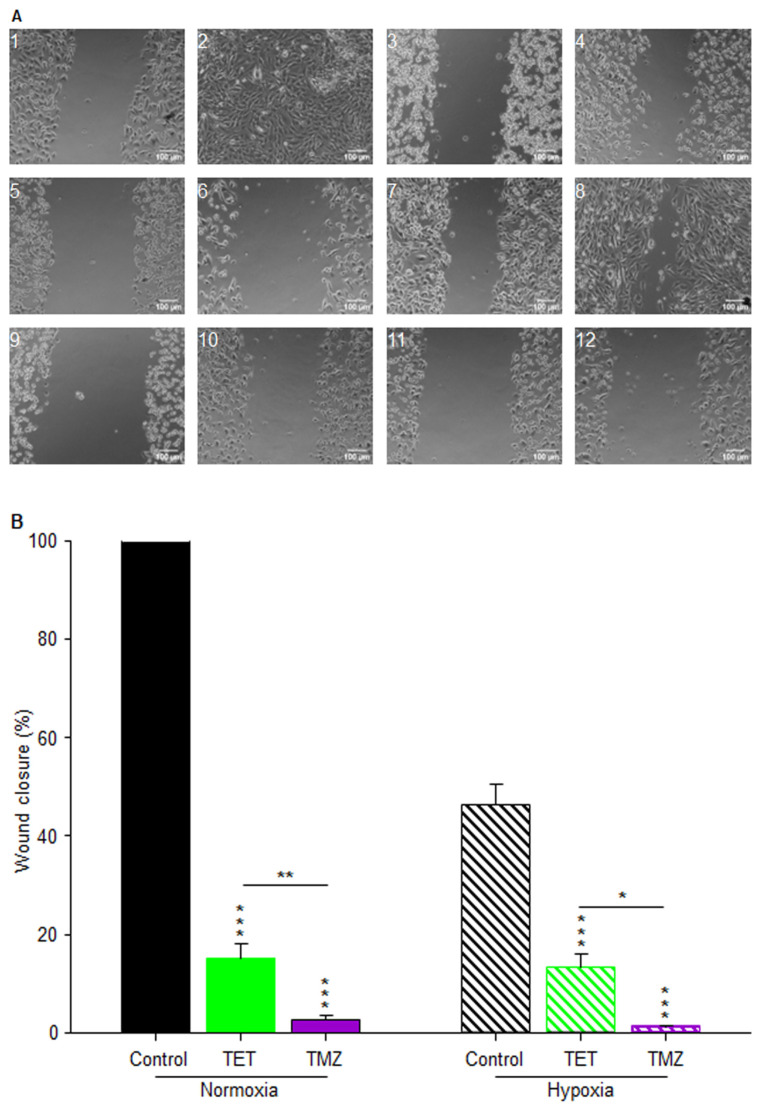
The scratch/wound healing assay of M010b cells following different treatments for 24 h under normoxic and hypoxic conditions. (**A**) Representative images at zero h and 24 h under normoxia [control cells (**A1**,**A2**) and cells treated with 1× IC_50_ TET (**A3**,**A4**) or TMZ (**A5**,**A6**)] and hypoxia [control cells (**A7**,**A8**) and cells treated with 1× IC_50_ TET (**A9**,**A10**) or TMZ (**A11**,**A12**)]. All photomicrographs were obtained at 10× magnification, and the scale bar indicates 100 µm. (**B**) Bar chart displays the percentage of wound closure in control cells and cells treated with 1× IC_50_ TET or TMZ under normoxic and hypoxic conditions. One-way ANOVA, followed by multiple comparisons versus the control, was performed to compare wound closure percentages in treated and control cells under normoxic and hypoxic conditions. Further analysis (One-way ANOVA followed by all pairwise multiple comparisons) was conducted to compare the percentage of wound closure in control cells, TET-treated cells, and TMZ-treated cells (horizontal lines with asterisks) under normoxia and hypoxia. * *p* ≤ 0.05, ** *p* ≤ 0.01, and *** *p* ≤ 0.001.

**Figure 15:**
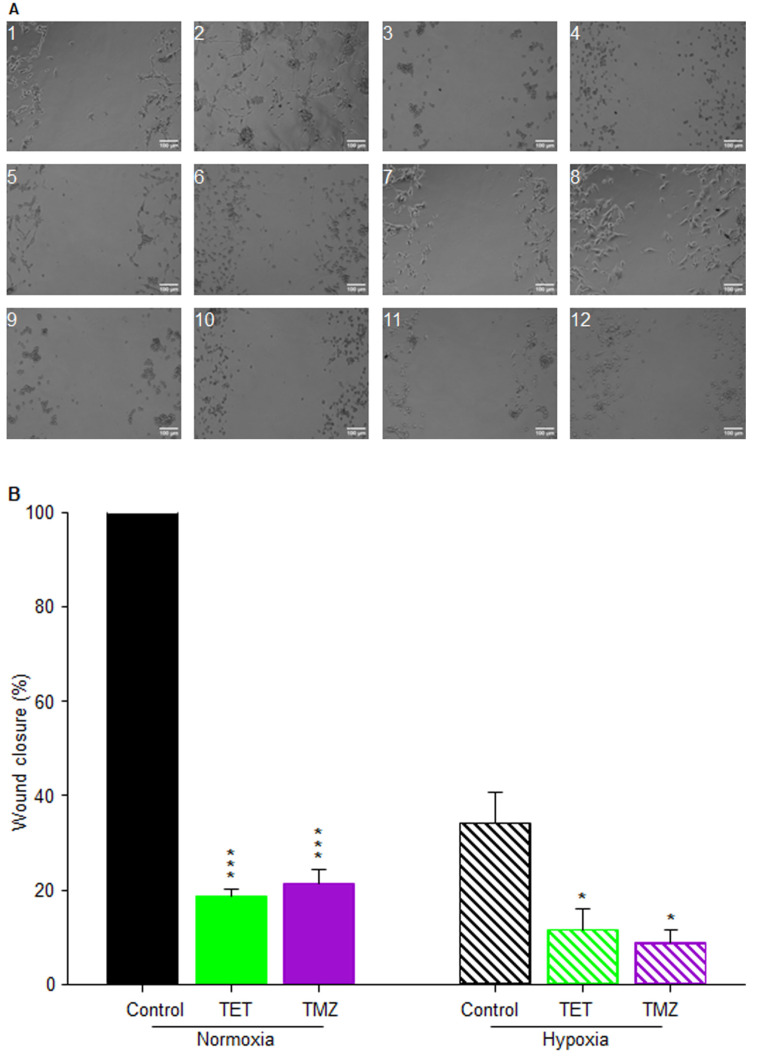
The scratch/wound healing assay of U87 cells following different treatments for 24 h under normoxic and hypoxic conditions. (**A**) Representative images at zero h and 24 h under normoxia [control cells (**A1**,**A2**) and cells treated with 1× IC_50_ TET (**A3**,**A4**) or TMZ (**A5**,**A6**)] and hypoxia [control cells (**A7**,**A8**) and cells treated with 1× IC_50_ TET (**A9**,**A10**) or TMZ (**A11**,**A12**)]. All photomicrographs were obtained at 10× magnification, and the scale bar indicates 100 µm. (**B**) Bar chart displays the percentage of wound closure in control cells and cells treated with 1× IC_50_ TET or TMZ under normoxic and hypoxic conditions. One-way ANOVA, followed by multiple comparisons versus control, was performed to compare the percentage of wound closure in treated and control cells under normoxic and hypoxic conditions. Further analysis (One-way ANOVA followed by all pairwise multiple comparisons) was conducted to compare the percentage of wound closure in control cells, TET-treated cells, and TMZ-treated cells (horizontal lines with asterisks) under normoxia and hypoxia. * *p* ≤ 0.05, and *** *p* ≤ 0.001.

To mimic cell migration in vivo, the in vitro scratch/wound healing assay was used to investigate the effects of TET and TMZ treatments on the migration ability of M010b and U87 cells. As shown in Figure 14, TET significantly decreased the migration capacity of M010b cells under normoxia and hypoxia, reducing it by 84.77% (Figure 14A(3,4),B) and 33.05% (Figure 14A(9,10),B), respectively. Similarly, in U87 cells (Figure 15), the migration capacity was significantly reduced by 81.29% (Figure 15A(3,4),B, under normoxia) and 22.56% (Figure 15A(9,10),B, under hypoxia). On the other hand, TMZ significantly inhibited the migration capacity in M010b cells by 97.3% (Figure 14A(5,6),B) and 44.93% (Figure 14A(11,12),B) under normoxic and hypoxic conditions, respectively. Additionally, in U87 cells (Figure 15), TMZ significantly decreased the migration capacity by 78.67% (Figure 15A(5,6),B, under normoxia) and 25.40% (Figure 15A(11,12),B, under hypoxia). Unlike U87 cells, TMZ caused a significantly greater reduction in migration capacity in M010b cells than TET (One-Way ANOVA, all pairwise multiple comparisons).

### 2.7. Docking Results Analysis

**Figure 16:**
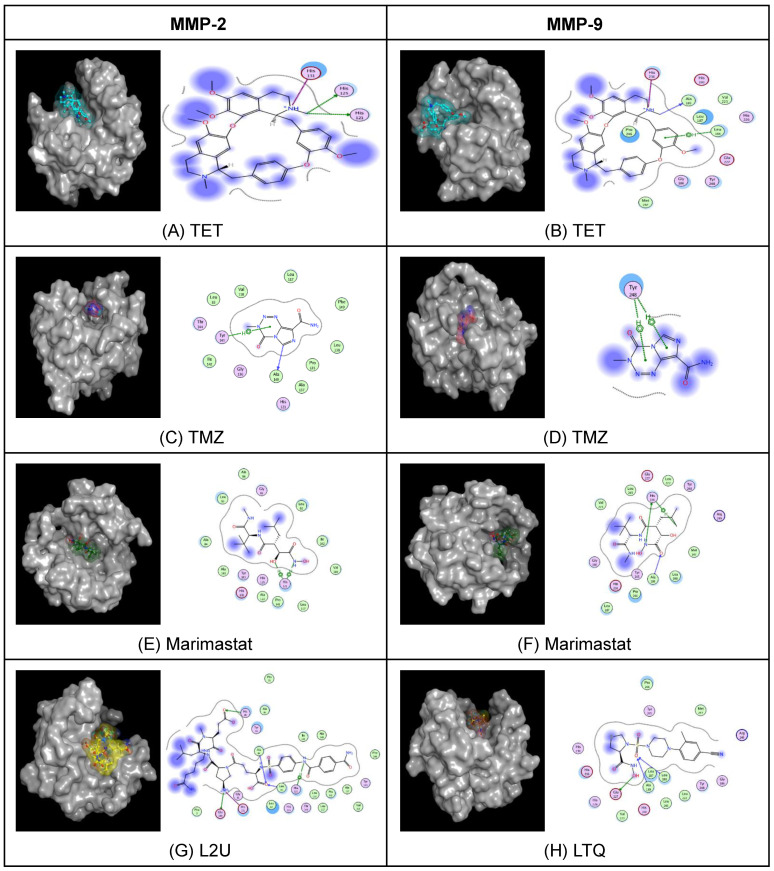
Three-dimensional and two-dimensional representations of TET (**A**,**B**), TMZ (**C**,**D**), marimastat (**E**,**F**), and co-crystals [L2U (**G**) and LTQ (**H**)] binding to MMP-2 and MMP-9.

MMP-2, MMP-9, TET, TMZ, and marimastat were selected for molecular docking using MOE 2014 software version 2014.0901. Generally, the active component shows strong binding to the target when the affinity score is less than −5.0 kcal/mol. The affinity scores of the docked TET inside the MMP-2 and MMP-9 pockets were −5.7 kcal/mol and −5.52 kcal/mol, respectively. TET formed two hydrogen bonds with HIS 121 and HIS 125 and one ionic bond with HIS 131 during its interaction with MMP-2. Additionally, the interaction of TET with MMP-9 resulted in one hydrogen bond with ALA 189, one ionic bond with HIS 236, and one pi-H bond with LEU 188. On the other hand, the docked TMZ in the MMP-2 pocket yielded a binding score of −4.6 kcal/mol, forming one hydrogen bond with ALA 140 and one Pi-H with TYR 143. The interaction of TMZ with MMP-9 pocket achieved a binding score of −4.98 kcal/mol, forming two Pi-H bonds with TYR 248. The binding scores for the docked marimastat in the MMP-2 and MMP-9 pockets were −6.0 kcal/mol and −6.6 kcal/mol, respectively. It formed two H-Pi bonds with HIS 121 in MMP-2 and two hydrogen bonds with ALA 189 and HIS 226, and one H-Pi bond with HIS 226 in MMP-9. These results showed that marimastat and TET exhibit better binding activities to MMP-2 and MMP-9 than TMZ. Additionally, TET showed binding modes like those of the co-crystallized ligands. Table 2 displays the binding scores and interactions with the pocket amino acids. The 2D and 3D interaction diagrams of TET, TMZ, marimastat, and the co-crystals with MMP-2 and MMP-9 are shown in Figure 16A–H.

### 2.8. TET and TMZ Reduced the Expression Levels of MMP-2 and MMP-9

**Figure 17:**
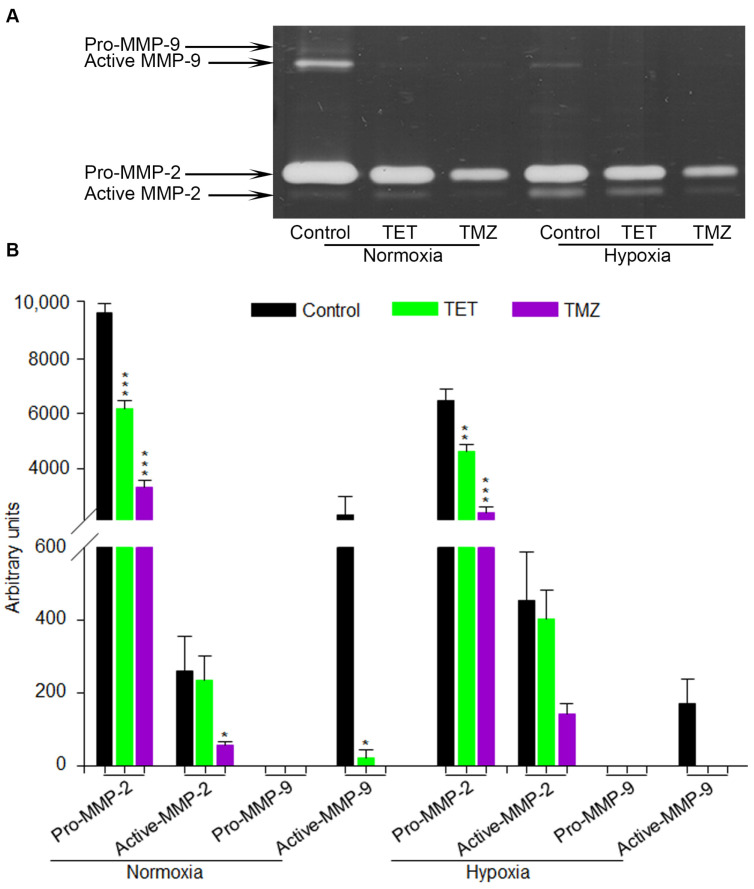
Representative zymogram (**A**) and levels of pro-MMP-2, active-MMP-2, pro-MMP-9, and active-MMP-9 (**B**) secreted by M010b cells treated with 1× IC_50_ TET and 1× IC_50_ TMZ for 24 h under normoxic and hypoxic conditions, alongside control cells. Pro-MMP-9 was undetectable. The *t*-test was used to compare active-MMP-9 in control and TET-treated cells under normoxia. One-way ANOVA, followed by multiple comparisons versus control, was performed to compare the pro-MMP-2, active-MMP-2, and active-MMP-9 levels in treated cells and their respective controls under normoxic and hypoxic conditions. * *p* ≤ 0.05, ** *p* ≤ 0.01, and *** *p* ≤ 0.001.

**Figure 18:**
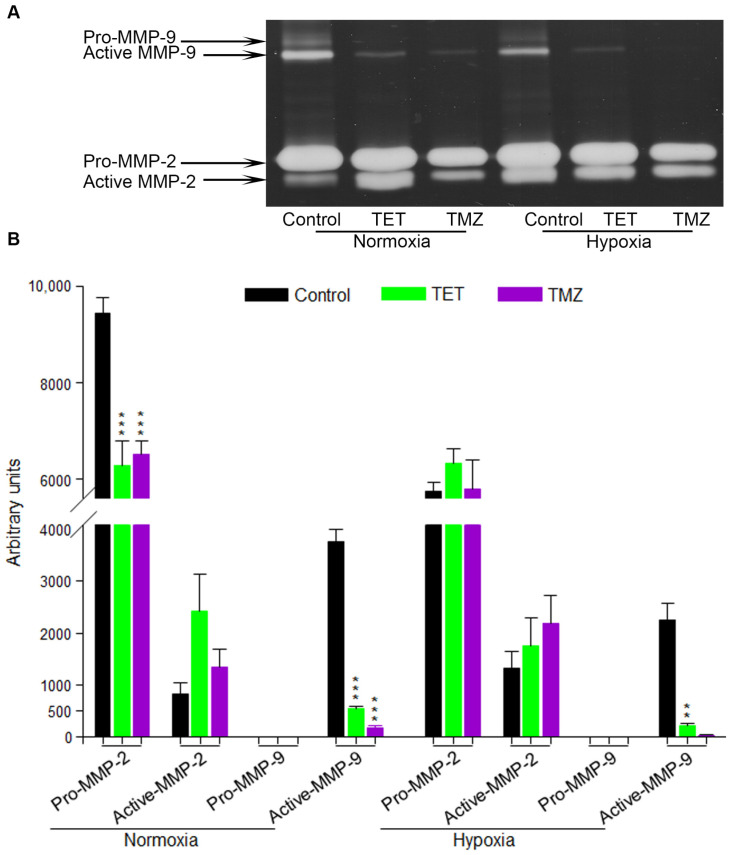
Representative zymogram (**A**) and levels of pro-MMP-2, active-MMP-2, pro-MMP-9, and active-MMP-9 (**B**) secreted by U87 cells treated with 1× IC_50_ TET and 1× IC_50_ TMZ for 24 h under normoxic and hypoxic conditions, alongside control cells. Pro-MMP-9 was undetectable. One-way ANOVA, followed by multiple comparisons versus control, was conducted to compare the pro-MMP-2, active-MMP-2, and active-MMP-9 levels in treated cells and their respective controls under normoxic and hypoxic conditions. ** *p* ≤ 0.01 and *** *p* ≤ 0.001.

MMP-2 and -9 play a crucial role in cancer cell migration, invasion, and metastasis by degrading type IV collagen, the major constituent of the basement membrane [44]. In parallel with the scratch assay, we investigated the effects of TET and TMZ on the contribution of MMP-2 and MMP-9 to the migration capacity of M010b and U87 cells using gelatin zymographic analysis. In M010b cells (Figure 17), under both normoxia and hypoxia, TET and TMZ significantly reduced the expression levels of pro-MMP-2, while under normoxia, the expression levels of active MMP-2 and active MMP-9 were significantly decreased by TMZ and TET, respectively. In U87 cells (Figure 18), under normoxia, the expression levels of pro-MMP-2 and active-MMP-9 were significantly reduced by either TMZ or TET, while under hypoxia, the active-MMP-9 was significantly reduced by TET.

## 3. Discussion

This study aimed to evaluate the therapeutic potential of TET, either in combination with or compared to TMZ, in treating GBM cells (M010b and U87) under both normoxic and hypoxic conditions. GBM is the most prevalent and deadliest type of brain tumor in adults [13] with a median survival rate of approximately 14.6 months [4]. TMZ is the standard chemotherapy for GBM and the only FDA-approved drug for treatment. TET has demonstrated several anti-cancer effects in multiple cancer cell lines [45]. Its mechanism of action involves inhibiting the proliferation, migration, and invasion, inducing apoptosis, and reversing multidrug resistance [45]. However, little is known about its effect on GBM cells.

In this study, we used CoCl_2_ to induce hypoxia in cell culture, where it elicits cellular, molecular, and biochemical responses similar to those occurring under hypoxia [46]. In U87 cells, it is reported that 100 μM CoCl_2_ induces hypoxia, and concentrations up to 100 μM do not significantly reduce viability [47]. However, a range of 100–600 μM CoCl_2_ is usually used to induce mimetic hypoxia in vitro [48]. Here, increasing CoCl_2_ concentrations gradually reduced cell viability and intensified hypoxia, as confirmed by hypoxia marker levels (VEGF, CAIX, LDHA, and GLUT1). Accordingly, 100 μM and 500 μM CoCl_2_ were used to induce hypoxia with minimal cytotoxicity in U87 and M010b cells, respectively. Although CoCl_2_ is commonly and widely used to study hypoxia-related cellular responses by mimicking low-oxygen-induced hypoxia, as it activates various hypoxia-driven genes, including apoptotic genes, glucose transporters, glycolytic enzymes, and cell cycle regulators, the transcriptional profile induced by CoCl_2_ does not fully match that of low-oxygen-induced hypoxia. Additionally, CoCl_2_ weakens the HIF-2α activity despite its stabilization, affects cell metabolism differently, and stimulates the activation of threonine, serine, and glycine pathways [38].

The sensitivity of GBM cells to TMZ treatment is based on their genetic makeup [49,50]. Lo Dico et al. reported a five-fold increase in TMZ concentrations required to induce significant reductions in cell viability in U87 and U251 cells under hypoxic conditions compared with normoxia [49]. Others confirmed a significant increase in the IC_50_ of TMZ under hypoxic conditions compared to normoxic conditions in U87, U251, and C6 cell lines [51,52]. Although this is the first study to investigate the effect of TET in GBM cells under hypoxia, few studies have shown its cytotoxic effect under normoxia [37,53,54]. TET and TMZ reduced the viability of M010b and U87 cells in a dose-dependent manner under normoxic and hypoxic conditions after 24 h of treatment. Interestingly, the IC_50_ values of TET under both conditions in both cell lines were significantly lower than those of TMZ (*p* ≤ 0.001, two-way ANOVA), indicating that GBM cells are significantly more sensitive to TET compared to TMZ. Additionally, unlike the response to TMZ, there were no significant differences between normoxic and hypoxic conditions in the responses of both cell lines to TET (*p* ≤ 0.01, three-way ANOVA). This may indicate a differential cellular response to TMZ. Notably, the high IC_50_ values of TMZ after 24 h exceeds the clinically achievable plasma levels as TMZ often results in delayed cytotoxic effect; however, the 24 h assessment of both drugs’ cytotoxic effects was selected to compare the early responses of cancer cells to both agents and ensure that the induced cytotoxicity is resulted from the actual potency of the drugs rather than the prolonged incubation of cells with the treatments. Combination therapy is more effective than monotherapy because it targets key pathways in a synergistic or additive fashion [55]. Therefore, we investigated whether TET could increase the sensitivity of GBM cells to TMZ. A synergistic effect was observed only under normoxia with combinations of TET and TMZ at concentrations of 2× and 4× IC_50_ in M010b and U87 cells, respectively. Additionally, an additive effect was seen with the combination of TET and TMZ at 0.25× IC_50_ in U87 cells under normoxic conditions. However, all other combinations under both conditions were antagonistic. As a result, only single treatments were used in this study.

The progression of the cell cycle is regulated by several checkpoints that evaluate cell size, growth signals, and DNA integrity. At the same time, its dysregulation is accompanied by uncontrolled cell proliferation and tumor development [56,57]. While the majority of reports show that TMZ arrests cells in G2/M phase in most cell lines [58,59,60], others reported a cycle arrest at the S phase [2,61,62] and a decrease in the percentage of cells arrested at G2/M [63]. Herein, in M010b cells, both 0.5× and 1× IC_50_ TMZ significantly increased the sub-G1 cell population under normoxia, while under hypoxia, they significantly arrested the cells in the G2/M phase. In U87 cells, 0.5× IC_50_ TMZ significantly increased the sub-G1 population under normoxia, and 1× IC_50_ TMZ significantly induced the highest G2/M phase arrest under both conditions, compared to other treatments. TET has been reported to induce cell cycle arrest in G0/G1 [37,64,65] and G2/M [66], and increase the percentage of cells in the sub-G1 region [24,25,67]. In this study, in M010b, TET at 0.5× IC_50_ and 1× IC_50_ significantly increased the sub-G1 population under both normoxic and hypoxic conditions. Additionally, 0.5× IC_50_ TET caused a significant increase in the G1 phase under normoxia. In U87 cells, a significant increase in the sub-G1 population was induced by 0.5× IC_50_ and 1× IC_50_ TET under normoxia and by 1× IC_50_ TET under hypoxia. In both cell lines and under both conditions, 1× IC_50_ TET significantly induced the highest increase in the sub-G1 population compared with other treatments. It is noteworthy that cycle arrest at different phases for a given effector is concentration- and time-dependent when the effector’s concentration or activity changes over time [68,69,70,71].

Anti-cancer agents primarily aim to induce apoptosis as a key strategy to eliminate cancer cells, since evasion of apoptosis is one of the hallmarks of cancer [29]. Here, in M010b cells, TMZ significantly increased early apoptotic, late apoptotic, and necrotic cells under normoxia, and late apoptotic cells under hypoxia. Additionally, it significantly decreased live cells under both conditions. However, in U87 cells, TMZ significantly increased early apoptotic cells under normoxia, with a non-significant effect under hypoxia. These results may suggest that TMZ has a greater therapeutic potential on M010b cells compared with U87 cells. These results are similar to those of previous reports on TMZ’s differential ability to induce apoptosis. Ge et al. have demonstrated that the ratio of apoptotic cells following TMZ exposure was decreased in hypoxic U87 cells compared to those under normoxic conditions, revealing that hypoxia lessens the induction of apoptosis by TMZ [51]. Lo Dico et al. reported that administering TMZ to normoxic sensitive and resistant cells resulted in a reciprocal Bax/Bad vs. Bcl-2 balance, similar to that observed in hypoxic cells. TMZ exposure reversed the hypoxia-induced expression pattern only in sensitive cells [49]. In other cancer cells, TMZ caused non-significant induction of apoptosis in U118 and GBM43 cells but significantly increased apoptosis in T98G cells [63,72]. Zhu et al. have shown that upon TMZ treatment for 72h, apoptosis was significantly induced in U373-RG0 and LN229-RG0 cells but to a much lesser extent in U373-RG2 and LN229-RG2 cells, where apoptosis-resistant cells, U373-RG2, were associated with an inhibition in caspase-3 signaling [73]. The differential response to U87 cells could be explained in the context of the specific cell’s genetic makeup and time-course treatment. On the other hand, a significant apoptotic response was observed following TET treatment. In both cell lines under both conditions, TET significantly decreased live cells and increased early apoptotic, late apoptotic, and necrotic cells. Similarly, Ma et al. have reported that 30 μg/mL TET caused apoptosis in 52.74% of U87 after 24 h [37]. In addition, TET upregulated the expression levels of caspase-3, cleaved caspase-9, total PARP, cleaved PARP, and Bax, suggesting a mitochondria-dependent apoptosis pathway [37]. Zhang et al. showed that TET treatment increased the apoptotic rate in a dose-dependent manner in U87 and U251 GSLCs by increasing GSK3β and Bax levels, promoting PARP cleavage, decreasing β-catenin levels, and inactivating Bcl-2 [74]. The effect of TET on apoptosis induction in GBM was further confirmed in an in vivo study by Liao et al. [26], where immunohistochemical analysis showed diminished levels of anti-apoptotic proteins MCL-1, c-FLIP, and XIAP and increased levels of the proapoptotic proteins cleaved caspase 3, caspase 8, and caspase 9. Notably, TET significantly caused the highest reduction in live cells in both cell lines under both conditions compared with TMZ.

Abnormally elevated ΔΨm in cancer cells is linked to enhanced metastases in vivo and increased invasive characteristics in vitro [75]. Herein, TMZ significantly decreased the ΔΨm in M010b and U87 cells under normoxia and hypoxia. However, this effect was lower under hypoxia (1.56-fold) versus normoxia (5.23-fold) in U87 cells. In agreement with our findings, Ge et al. stated that TMZ-treated hypoxic cells showed less mitochondrial depolarization than TMZ-treated normoxic cells, suggesting the role of hypoxia in the preservation of mitochondrial function and the reduction in the induction of apoptosis [51]. In contrast, TMZ showed no effect on the ΔΨm in U251 cells [76]. On the other hand, this study showed that TET significantly decreased the ΔΨm in M010b cells under both normoxic and hypoxic conditions. In line with this result, TET decreased the ΔΨm in colon, pancreatic, and oral cancer cell lines [25,77,78]. Whereas TET showed no significant change in the ΔΨm levels in nasopharyngeal carcinoma cells [67].

There is controversy over ROS’s contribution to carcinogenesis. ROS acts as signaling molecules that enhance the proliferation and differentiation of cells when their levels are low or moderate, while at high levels, ROS induces cell death; thus, several chemotherapeutics work as inducers of ROS generation in cancer cells [29]. In this study, TET significantly triggered the production of ROS in M010b and U87 cells under normoxic conditions. In contrast, TET had no effect under hypoxic conditions. This may suggest that hypoxia impaired the ability of TET to induce significant ROS production. In agreement with our findings, TET was reported to elevate the levels of ROS in vitro in various types of cancer, including gastric cancer, colon cancer, nasopharyngeal carcinoma, liver cancer, and pancreatic cancer [29,67,78,79]. These studies reported that pretreatment of different cancer cell lines with ROS scavengers eliminated TET-induced ROS generation and, in turn, prevented cell death, suggesting that the increase in the levels of ROS contributes to the induction of apoptosis caused by TET. On the other hand, our findings showed that TMZ did not stimulate the generation of ROS under both conditions in both cell lines. Meanwhile, it significantly lowered ROS production in normoxic and hypoxic M010b cells and normoxic but not hypoxic U87 cells. Notably, TET significantly caused the highest induction in ROS in both cell lines under both conditions compared with TMZ. In contrast to our findings, previous studies documented that TMZ elevates ROS production in glioma cell lines T98G, U87, U251, LN229, and SHG44 cells, but not U118, UTMZ cells [72,80,81,82,83]. The discrepancy between our findings and previous studies may be interpreted in the context of the duration of treatment [84,85].

Migration and invasion mechanisms enable cancer cells to metastasize from a primary tumor site to potential metastatic sites via lymphatic and blood vessels [86]. MMPs play a pivotal role in this process; they catalyze the degradation of cell adhesion molecules, growth factor-binding proteins, extracellular matrix proteins, receptor tyrosine kinases, growth factor precursors, and other proteases, thereby facilitating cancer cell invasion [87]. Additionally, upregulation of MMP expression contributes to tumor cell proliferation, evasion of apoptosis, and inhibition of antitumor immune surveillance [87]. Higher glioma grades are often associated with overexpression of MMP-2 and MMP-9; thus, inhibiting these enzymes reduces glioma cell invasiveness and could serve as a promising therapeutic strategy [88]. Here, we report that both TET and TMZ significantly reduced the migration capacity of both cell lines under normoxia and hypoxia. The molecular docking analysis showed that TET has a strong binding affinity for MMP-2 and MMP-9. We experimentally verified the molecular docking study by gelatin zymographic analysis. TET and TMZ significantly decreased the expression levels of pro-MMP-2 and active-MMP-9 in both cell lines under normoxia. In M010b cells, both drugs significantly reduced pro-MMP-2 expression and modestly decreased active-MMP-2 under hypoxia. In line with these results, Wang et al. demonstrated that the migration and invasion of glioma C6 cells were significantly suppressed upon TMZ treatment [89]. In addition, Mirabdaly et al. confirmed that TMZ reduced the MMP-2 and MMP-9 activities in U87 cells [90]. Whereas Suzuki et al. showed that TMZ treatment for 24 or 48 h did not affect MMP-2 or MMP-9 secretion in A172 or U373MG cells [91]. Wen et al. showed that TMZ partially inhibited U251 cell migration after 12 h [92]. TET reduced cell migration and invasion in various GBM cell lines, including U87, GBM 8401, U87 GSLCs, and U251 GSLCs [53,54,74]. Additionally, it suppressed the MMP-2 gelatinolytic activity in GBM 8401 cells after 48 h, but not after 24 h of treatment [53]. Further studies investigating the potential inhibitory effect of TET on heparanase, another enzyme involved in cancer invasion and metastasis, autophagy induction, and poor prognosis in GBM [93], are warranted.

The observed reduction in cancer cell viability, induction of apoptosis, increased ROS production, mitochondrial depolarization, and decreased migration capacity indicate that TET can affect multiple survival pathways. Luan et al. summarized that TET-induced anticancer effects can be mediated through the regulation of the following signaling pathways: PI3K/AKT, AMPK/mTOR, PI3K/AKT/mTOR, Hippo/YAP, MAPK, caspases/beclin I/LC3-I/II, ERK/MAPK, PARP, death receptor, Wnt/cadherin, and Wnt/β-catenin signaling [45]. Since lipid rafts have been reported to play a key role in tissue plasminogen activator-stimulated signaling involving β-catenin [94], the induced anticancer effects of TET may be associated with alterations in lipid raft-associated signaling and warrant further investigation.

This study had some limitations that should be addressed in our future work, including assessing TET’s selectivity and safety profile in normal glial cells to evaluate cytotoxicity, and conducting in vivo studies to validate TET’s translational and therapeutic potential in GBM.

## 4. Materials and Methods

### 4.1. Cell Lines and In Vitro Culture Conditions

The origin and characterization of the GBM cell lines have been published previously: M010b cell line [95,96,97,98,99]; the U87 MG-HTB-14 cell line was purchased from the American Type Culture Collection (ATCC, VA, USA). Cells were cultured in DMEM–F12 with Stable Glutamine and 15 mM Hepes (Cat. No. L0092-500 Biowest, Nuaillé, France), supplemented with 10% fetal bovine serum (FBS) (Cat. No. S1810–500 Biowest, Nuaillé, France). The cells were grown in a humidified incubator at 37 °C and 5% CO_2_ and passaged at 70–80% confluence. No antibiotics were added [100]. The U87 and M010b cells were used at passage numbers less than 40. Cell culture plasticware was purchased from SPL, Life Sciences, Pocheon-si, Republic of Korea. Hypoxia was induced using cobalt chloride (CoCl_2_, Cat. No. C/6520/48, Fisher Scientific, Altrincham, UK). Stock solutions of Tetrandrine (Cat. No. QB-6517, Combi Blocks, San Diego, CA, USA) and Temozolomide (Cat. No. QB-2567, Combi Blocks) were prepared in dimethyl sulfoxide (DMSO, Cat No. 67-68-5, Sisco Research Laboratories Pvt. Ltd., New Mumbai, India). Stock solutions were aliquoted and stored at −20 °C. Each stock solution was diluted to the required concentration in complete growth medium (DMEM F-12).

### 4.2. Reverse Transcription-Quantitative Polymerase Chain Reaction (RT-qPCR)

Total RNA was extracted using the RNeasy Mini Kit (Cat. No. 74136, Qiagen, Hilden, Germany). Reverse transcription was performed according to the manufacturer’s instructions using the high-capacity cDNA Reverse Transcription Kit (Cat. No. 4368814; Applied Biosystems, Thermo Fisher Scientific, Inc., Waltham, MA, USA) with RNase Inhibitor (Cat. No. N8080119; Applied Biosystems, Thermo Fisher Scientific, Inc.). RT-qPCR analysis was performed using a Quant Studio 12K Flex Real-Time PCR System (Applied Biosystems; Thermo Fisher Scientific, Inc.). TaqMan Fast Universal PCR Master Mix (cat. no. 4366072; Applied Biosystems; Thermo Fisher Scientific, Inc.) and validated TaqMan gene expression assays (Applied Biosystems; Thermo Fisher Scientific, Inc.) were used for CAIX (Hs00154208_m1), GLUT-1 (Hs00892681_m1), LDHA (Hs01378790_g1), and VEGF (Hs00900055_m1). The human β-actin gene (assay no Hs99999903_m1) was used as an endogenous housekeeping control. Using the 2^−ΔΔCT^ method [101], the fold change in the target gene expression, normalized to the endogenous housekeeping gene and relative to control, was quantified.

### 4.3. Cell Viability and Proliferation Assay

Cell viability and proliferation were assessed using 3-(4, 5-dimethylthiazol-2-yl)-2, 5-diphenyltetrazolium bromide (MTT) (Cat. No. 20395.02, Serva, Germany). GBM cells (12,500 cells per well) were seeded in triplicate in 96-well plates and incubated for 24 h. The cells were treated with TET concentrations (10–100 µM) or TMZ concentrations (100–5000 µM) under normoxic or hypoxic conditions for 24 h. Afterward, the drug-containing medium was aspirated and replaced with 100 µL of MTT solution (0.5 mg/mL in culture medium). Cells were incubated for 4 h, then the MTT solution was removed, and 100 µL of DMSO per well was added and left for 15 min. The optical density was measured at 570 nm using a microplate reader (FLUOstar Omega, BMG LABTECH GmbH, Ortenberg, Germany). The IC_50_ values of the tested drugs under normoxic and hypoxic conditions were estimated using GraphPad Prism version 8.4.0 (GraphPad Software, Boston, MA, USA).

### 4.4. Evaluation of Drug Interactions

The MTT assay was performed as described above to assess growth inhibition caused by TET, TMZ, and their combination. Briefly, GBM cells were cultured in 96-well plates (12,500 cells per well). After 24 h, the cells were treated with TET, TMZ, and the combination of TET and TMZ at increasing concentrations (0.25×, 0.5×, 1×, 2×, and 4× IC_50_) under both normoxic and hypoxic conditions. Following treatment, cell viability was measured under both conditions, and the combination index (CI) was determined [39] using CompuSyn software version 1.0 (ComboSyn, Inc., Paramus, NJ, USA), where CI values of <1, >1, and 1 indicate synergism, antagonism, and additivity, respectively.

### 4.5. Cell Cycle Analysis

Cell cycle analysis was done as previously described [102] with slight modifications. The GBM cells were treated with 0.5× and 1× IC_50_ of TET or TMZ for 24 h under normoxic and hypoxic conditions. The cells were trypsinized, washed with cold PBS, fixed in 70% ethanol, and incubated on ice for 30 min. The cells were washed, suspended in staining buffer [PBS with 100 μg/mL RNase A (Cat. No. 10109142001, Sigma Aldrich, St. Louis, MO, USA) and 50 μg/mL of propidium iodide (Sc-3541, Santa Cruz Biotechnology, Dallas, TX, USA)] and incubated for 30 min in the dark at room temperature. The stained nuclei were examined using the CytoFlex^®^ flow cytometer (Beckman Coulter, Brea, CA, USA).

### 4.6. Apoptosis Analysis

Using the dead cell apoptosis kit [Alexa Fluor 488 Annexin V and propidium iodide (PI), Cat. No. V13245, Thermo Fisher Scientific, Inc.], early apoptosis, late apoptosis, and necrosis were determined according to the manufacturer’s instructions. In brief, GBM cells were seeded at a density of 200,000 cells per well in 6-well plates. At 60–70% confluency, the cells were treated with the 1× IC_50_ of TET or TMZ for 24 h under both normoxic and hypoxic conditions. Afterward, the cells were harvested and washed with cold PBS. Each sample was washed and resuspended in 1X annexin-binding buffer, where the cell density was adjusted to 10^5^ cells per 100 µL. Then, 5 µL of Alexa Fluor 488 Annexin V and 1 µL of propidium iodide (100 μg/mL) were added to each sample. The samples were incubated in the dark at room temperature for 15 min. Following incubation, 400 µL of 1X annexin-binding buffer was added. The samples were gently mixed and kept on ice. Finally, the samples were analyzed using a CytoFlex^®^ flow cytometer.

### 4.7. Mitochondrial Membrane Potential Analysis

Alterations in the mitochondrial membrane potential (ΔΨm) were measured by JC-1 staining (Cat. No. sc-364116, Santa Cruz Biotechnology, Dallas, TX, USA). The GBM cells were cultured in 96-well F-bottom (Chimney well) plates (Cat. No. 655090, Greiner Bio-One GmbH, Frickenhausen, Germany). When the cells reached 60–70% confluence, they were treated with 1× IC_50_ of TET or TMZ for 24 h under normoxic and hypoxic conditions. After treatment, the cells were stained with one µM JC-1 solution (100 µL/well) and incubated at 37 °C in the dark for 30 min. The treated and control cells were washed twice with PBS containing TET or TMZ and PBS, respectively, and then left in the last wash in the wells. Subsequently, the cells were analyzed using a FLUOstar Omega microplate reader at excitation 544 nm/emission 590 nm for JC-1 aggregate detection and at excitation 485 nm/emission 520 nm for JC-1 monomer detection, where the ratio of monomer/aggregate (green/red) fluorescence was calculated relative to the control.

### 4.8. Reactive Oxygen Species (ROS) Detection

The cell-permeable fluorescent probe CM-H2DCFDA (Cat. No. C6827, Thermo Fisher Scientific, Inc.) was used to assess intracellular ROS levels. GBM cells (15,000 cells per well) were cultured in 96-well F-bottom (Chimney well) plates. When reaching 60–70% confluency, the cells were treated with 1× IC_50_ of TET or TMZ for 24 h. Afterward, the cells were washed with PBS and incubated with 10 µM CM-H2DCFDA (100 µL per well) for 30 min in the dark at room temperature. Subsequently, the cells were washed twice with PBS, and the fluorescence signals were measured at excitation/emission wavelengths of 485 nm and 520 nm, respectively, using the microplate reader.

### 4.9. Scratch/Wound Healing Assay

The GBM cells (200,000 cells per well) were cultured in 24-well plates. At approximately 90% confluency, the cell monolayers were scraped with 10 µL sterile pipette tip to create a single-line wound. The cells were then washed with PBS and treated with the IC_50_ of TET or TMZ in DMEM-F12 medium supplemented with 1.5% FBS under both normoxic and hypoxic conditions. The wound area was photographed at 0 and 24 h using the inverted microscope (Leica DMi1, Wetzlar, Germany). The wound width was quantified using the ImageJ software version 1.54g (NIH, Bethesda, MD, USA). The cell migration inhibition rate (%) was calculated as: (original scratch width—final scratch width)/original scratch width × 100.

### 4.10. Molecular Docking

The 3D structures of matrix metalloproteinase-2 (MMP-2) and MMP-9 were obtained from the Protein Data Bank (PDB IDs: 8H78 for MMP-2 and 5CUH for MMP-9) and prepared using the Molecular Operating Environment (MOE) structure preparation wizard. The structures of TET, TMZ, and marimastat (broad-spectrum MMP inhibitor [103]) were drawn using ChemDraw software version 16.0.0.82 (68) (PerkinElmer, Waltham, MA, USA). After applying the lowest-energy conformation of compounds and removing the co-crystallized ligands L2U and LTQ from MMP-2 and MMP-9, respectively, TET, TMZ, and marimastat were docked into their binding sites using MOE 2014 software (Chemical Computing Group, Montreal, QC, Canada). The poses with the best RMSD-refined values, binding scores, and binding modes similar to the co-crystallized ligands were chosen for further analysis.

### 4.11. Gelatin Zymographic Analysis

Matrix metalloproteinases (MMPs): Pro-MMP-2, active-MMP-2, pro-MMP-9, and active-MMP-9 secreted into the conditioned medium were quantified using gelatin zymographic analysis [97]. The GBM cells (500,000 cells) were seeded in 6 cm plates in complete growth medium. At 60–70% confluency, the cells were washed twice with PBS, treated with the 1× IC_50_ values of TET or TMZ under normoxic and hypoxic conditions, and incubated in serum-deprived medium for 24 h. Afterward, the medium was collected and centrifuged for 5 min at 900 rpm to remove all cellular debris, then stored at −80 °C until analysis. Equal media samples were mixed with 1× sample buffer and separated on a 10% SDS-polyacrylamide gel containing 0.2% gelatin (Cat. No. G1890-100G, Sigma, St. Louis, MO, USA). Following electrophoresis, the gels were washed three times in 2.5% Triton X-100 at room temperature for 20 min each to remove SDS. The gels were then incubated in an incubation buffer (50 mM Tris/HCl, 10 mM CaCl_2_, 200 mM NaCl, 0.05% NaN_3_, pH 7.5) at 37 °C for 24 h. Gels were stained using 0.05% Coomassie brilliant blue G-250 (Cat. No. 27815-25G-F, Sigma), 25% methanol, and 10% acetic acid. They were shaken for two h at room temperature and then destained at room temperature in 4% methanol and 8% acetic acid and shaken for two h. The gels were scanned (CanoScan LiDE 700F, Canon Inc., Tokyo, Japan), and the gelatinolytic bands of MMP-2 and MMP-9 were analyzed using ImageJ software (NIH, USA). The activity of the gelatinolytic bands was expressed in arbitrary units.

### 4.12. Statistical Analysis

The data is presented as the mean ± SE of three experimental replicates. SigmaPlot software version 13.0 (Systat Software Inc., San Jose, CA, USA) was used to perform *t*-test, one-way Analysis of Variance (ANOVA), one-way ANOVA on Ranks, followed by multiple comparisons versus control or all pairwise multiple comparisons, two-way ANOVA, and three-way ANOVA. Asterisks indicate a significant difference (* *p* ≤ 0.05, ** *p* ≤ 0.01, and *** *p* ≤ 0.001).

## 5. Conclusions

In conclusion, in M010b and U87 cells, under both normoxic and hypoxic conditions, TET significantly reduced cell viability and proliferation, compared to TMZ. It also exhibited significant inhibitory effects on cell cycle progression, cell migration, and MMPs secretion, and significantly increased apoptosis and ROS production. Given the limited therapeutic potential of TMZ, our findings suggest that TET could serve as an alternative treatment for GBM; however, further mechanistic, pharmacokinetic, and in vivo studies are needed to validate its therapeutic potential.

## Figures and Tables

**Table 1:** The IC_50_ (µM) values of TET and TMZ in M010b and U87 cells under normoxia and hypoxia after 24 h.

Cell Line	Drug (μM)	Normoxia	Hypoxia
M010b	TET	31.08 ± 1.68	28.315 ± 1.8
	TMZ	1737.67 ± 152.1	2227 ± 90.5
U87	TET	32.57± 2.08	41.44 ± 2.6
	TMZ	2254.3 ± 122.31	2315.67 ± 283.52

**Table 2:** Docking binding free energies and interactions of TET, TMZ, marimastat, and co-crystals (L2U and LTQ) with MMP-2 and MMP-9.

Macromolecule	Drug	Ligand	Receptor	Interaction	Distance (Å)	E (Kcal/Mol)	S (Energy Score)
MMP2	TET	C 66	NE2 HIS 121	H-donor	3.4	−1	−5.7
		C 66	NE2 HIS 125	H-donor	3.53	−0.7	
		N 12	NE2 HIS 131	Ionic	3.93	−0.7	
	TMZ	C 11	O ALA 140	H-donor	3.2	−1.2	−4.6
		6-ring	CA TYR 143	Pi-H	4.04	−1.3	
	Marimastat	N 8	5-ring HIS 121	H-Pi	4.03	−0.8	−6.0
		O 26	5-ring HIS 121	H-Pi	3.63	−1.0	
	L2U	N 84	OE GLU 130	H-donor	2.73	−17.4	−10.3
		O 13	N LEU 83	H-acceptor	2.95	−3	
		O 13	N ALA 84	H-acceptor	3.03	−0.7	
		O 62	ND1 HIS 85	H-acceptor	3.22	−1.6	
		N 84	OE1 GLU 130	Ionic	3.48	−2.0	
		N 84	OE2 GLU 130	Ionic	2.73	−6.6	
		N 84	ND1 HIS 131	Ionic	3.9	−0.7	
		N 103	5 ring HIS 121	H-Pi	3.79	−0.6	
MMP9	TET	C 66	O ALA 189	H-donor	3.22	−1	−5.52
		N 12	NE2 HIS 236	Ionic	3.99	−0.5	
		6-ring	CD1 LEU 188	Pi-H	4.22	−0.8	
	TMZ	6 ring	CA TYR 248	Pi-H	4.67	−1.0	−4.98
		5 ring	CA TYR 248	Pi-H	4.57	−1.0	
	Marimastat	N 8	NE2 HIS 226	H-donor	3.45	−1.3	−6.6
		O 7	N ALA	H-acceptor	3.35	−0.8	
		C 22	5-ring HIS 226	H-Pi	3.89	−0.8	
	LTQ	O 15	OE2 GLU 227	H-donor	3.03	−3.9	−7.48
		O 18	N LEU 188	H-acceptor	3.39	−1.4	
		O 18	N ALA 189	H-acceptor	3.12	−2.7	

## Data Availability

The data supporting the findings of this study are available from the corresponding author upon reasonable request.

## Abbreviations

The following abbreviations are used in this manuscript:

AKT	protein kinase B
AMPK	AMP-activated protein kinase
ATCC	American type culture collection
Bad	Bcl-2-associated agonist of cell death
BAX	Bcl-2-associated X protein
Bcl-2	B-cell lymphoma 2
CAIX	Carbonic anhydrase
c-FLIP	Cellular FLICE-like inhibitory protein
CI	Combination index
DMEM F-12	Dulbecco’s modified eagle medium/nutrient mixture F-12
DMSO	Dimethyl sulfoxide
DNA	Deoxyribonucleic acid
ERK	extracellular signal-regulated kinase
Fa	Fraction affected
FBS	Fetal bovine serum
GBM	Glioblastoma multiforme
GLUT-1	Glucose transporter 1
HIF-1	Hypoxia-inducible factor 1
HREs	Hypoxia responsive elements
IC_50_	Half maximal inhibitory concentration
LDHA	Lactate dehydrogenase A
MCL-1	Myeloid cell leukemia-1
MGMT	O^6^-methylguanine-DNA-methyltransferase
MMP	Matrix metalloproteinases
MOE	Molecular operating environment
mTOR	mammalian target of rapamycin
MTT	3-(4, 5-dimethylthiazol-2-yl)-2, 5-diphenyltetrazolium bromide
PARP	Poly (ADP-ribose) polymerase
PBS	Phosphate-buffered saline
PDB	Protein data bank
PI	Propidium iodide
PI3K	phosphoinositide-3-kinase
qRT-PCR	Reverse transcription-quantitative polymerase chain reaction
RMSD	Root-mean-square deviation
RNA	Ribonucleic acid
ROS	Reactive oxygen species
SDS	Sodium dodecyl sulfate
TET	Tetrandrine
TMZ	Temozolomide
VEGF	Vascular endothelial growth factor
WHO	World health organization
Wnt	Wingless
XIAP	X-linked inhibitor of apoptosis protein
YAP	Yes associated protein
